# Developing genetic literacy in high school students with intellectual disability: Teachers’ experiences and perspectives

**DOI:** 10.1038/s41431-025-01865-2

**Published:** 2025-06-06

**Authors:** Karen-Maia Jackaman, Iva Strnadová, Sierra Angelina Willow, Julie Loblinzk Refalo, Jackie Leach Scully, Elizabeth Emma Palmer, Bronwyn Terrill

**Affiliations:** 1 School of Education, UNSW Sydney, Sydney, NSW Australia; 2 Disability Innovation Institute, UNSW Sydney, Sydney, NSW Australia; 3 Self-Advocacy Sydney, Sydney, NSW Australia; 4 School of Paediatrics and Child Health, Faculty of Medicine and Health, UNSW Sydney, Sydney, NSW Australia; 5 Sydney Children’s Hospitals Network, NSW Health, Sydney, NSW Australia; 6 Garvan Institute of Medical Research, Darlinghurst, NSW Australia; 7 School of Clinical Medicine, Faculty of Medicine and Health, UNSW Sydney, Sydney, NSW Australia

**Keywords:** Genetics, Patient education

## Abstract

Genetics is a rapidly evolving field with the potential to achieve improved health outcomes through precision medicine. People with intellectual disability have asked to know more about genetic conditions that they may have, but require education to build their genetic literacy, thereby empowering them to make informed healthcare decisions. Key to this is ensuring students with intellectual disability can access and participate in genetics education at school. Despite integration of genetics into curriculum, little is known about whether teachers are equipped to engage students with intellectual disability with this content. To explore this issue, fifteen teachers who teach genetic content to students with intellectual disability participated in semi-structured interviews or in a focus group. The analysis revealed three interconnected themes addressing genetics education for students with intellectual disability. These themes encompassed effective pedagogical approaches and curriculum adaptations, the necessity for targeted professional development with appropriate resources, and the importance of fostering comprehensive genetic literacy across the entire school community to build capacity among students, staff, and families. These findings have widespread implications for supporting teachers to develop genetic literacy in students with intellectual disability. A key recommendation is to create professional learning and a suite of accessible, multimodal, online resources for students with intellectual disability, their teachers, and the broader school community.

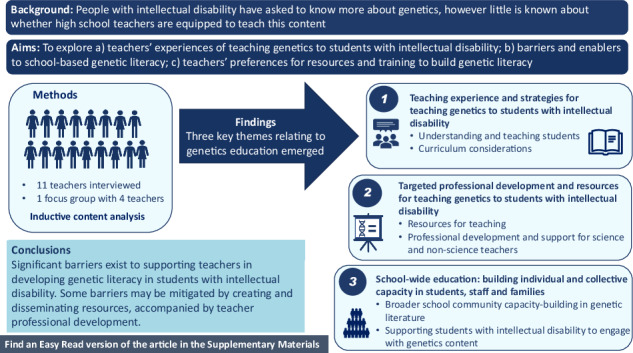

## Introduction

People with intellectual disability have the right to accessible information about healthcare and support to make healthcare choices [1, 2] and have voiced desire to learn more about their own genetic make-up and condition(s) [3–5]. Recent Australian research demonstrated that adults with intellectual disability wished that they had learnt more about genetics at school, to better understand their own genetic conditions and make informed healthcare decisions [3].

For many people with and without intellectual disability, schools provide their only formal opportunity to learn about science and genetics. Students with intellectual disability can and should learn science content in accessible ways [6, 7]. Science learning develops higher-order skills such as problem-solving and communication [6] reducing dependence on adults and supporting decision-making [8]. Science class is also where most students learn about genetics, with genetic content in most national curricula [9, 10], thus secondary schools are well placed to offer foundational genetics knowledge through science syllabuses [11].

If successful, schools can help students with intellectual disability learn about genetics and build skills to make informed choices about genetic testing and medicine (i.e. their genetic health literacy). Schools can also help students better understand the information that health professionals discuss with them. Understanding basic genetic information when making health decisions has potential, alongside more inclusive genetic-related healthcare, to improve access to therapies and support for people with intellectual disability, potentially reducing recognised health and wellbeing gaps relative to the general population [12, 13]. A key challenge in genetic education is tailoring abstract and complex biological concepts, with highly specialised vocabulary, for diverse audiences [10, 14]. Researchers concluded that most science teachers lack both knowledge to teach genetics [15] and accessible, differentiated resources to meet diverse learners’ needs [11, 16].

Teaching genetics is particularly challenging for students with intellectual disability, who have limited conceptual, social, and practical functioning [17, 18]. Academic challenges include issues with working memory, sustained attention, use of abstract concepts, and processing speed [18]. Teachers often lack comprehensive understanding of evidence-based practices for teaching students with disability [19–21]. For example, task analytic instruction and time delay have been proven effective both for teaching science content [6] and improving academic outcomes for students with intellectual disability [19, 22, 23]. Other evidence-based practices include graphic organisers [19, 24], manipulatives [19, 23, 25], visual supports [19, 26, 27] and peer support arrangements [19, 28]. Further information appears in Supplementary Table 1.

## Context of the study

This study was conducted in New South Wales (NSW), Australia. The national *Disability Standards for Education 2005* (DSE) mandate reasonable adjustments (modifications to learning environment, materials, or teaching methods to enable equal participation - also called ‘accommodations’ or ‘access arrangements’) and parent consultation to ensure that students with disability can participate in education on the same basis as students without disability [29]. If these adjustments prove insufficient, high school students with intellectual disability can access ‘Life Skills’ courses which align with mainstream content and outcomes (see Supplementary Table 2) [30]. NSW high school science syllabuses mandate teaching genetics-related content [31–33]. Staff shortages [34, 35] have led to staff teaching science beyond their subject expertise, increasing workload and stress [36] - particularly when teaching complex topics like genetics without specialised training or appropriate resources [37]. Students with intellectual disability in NSW specialist schools or classes are often taught by teachers qualified in ‘special and inclusive education’ rather than science [34, 38, 39] as they teach all subject areas. This lack of subject matter expertise heightens challenges in teaching genetics to students with intellectual disability; however, all study participants, regardless of qualifications, should be teaching genetic-related content from NSW high school science syllabuses [31–33] (See Supplementary Tables 2–4).

Research shows low levels of teacher confidence in differentiating for students with disability [40] and inconsistent confidence teaching genetic content [41] with teachers feeling under-skilled and under-resourced [42]. However, to our knowledge no studies have explored teachers’ confidence in teaching genetics to students with intellectual disability. This study aimed to explore (a) teachers’ experiences of teaching genetics to students with intellectual disability, (b) barriers to and enablers of school-based genetic literacy for students with intellectual disability, and (c) teachers’ preferences for resources and training.

An Easy Read version of this paper is available in the Supplementary Information.

## Materials and methods

### Research design

This study used qualitative research design to facilitate deep understanding of participants’ thoughts, experiences and feelings [43]. Similar qualitative research into teachers’ experiences has used semi-structured interviews [44, 45] that enable structured exploration of key areas, indicating appropriate methodology. This design enabled authors to engage deeply with participants’ voices and provided data for in-depth analysis of how genetic concepts are being taught, challenges faced, and resources needed. Interviews and focus groups were selected to generate qualitative data about participants’ views, experiences and beliefs [46], and accommodate preferences in engagement. Semi-structured questions provided flexibility to probe deeper or tailor questions based on responses [47, 48].

### Participants

Convenience sampling recruited fifteen teachers who taught science to high school students with intellectual disability in NSW schools. Inclusion/exclusion criteria and recruitment information appear in Supplementary Tables 5 and 6. Participants included eleven women and four men, aged 28–62 years (average 45). They taught in mainstream schools, support classes within mainstream schools, and specialist schools for students with disability. Teaching experience averaged 16 years (range 2–36 years). Eleven had tertiary qualifications in science and four in special and inclusive education. One had both science and special education qualifications, and one had neither. Demographic details appear in Table 1.

**Table 1:** Participant demographic data.

Name (pseudonym)	Age	Gender	Relevant qualifications	Years of teaching	Settings and role in teaching science
Candice	49	Female	Post graduate diploma in Science (Honours in Biology)	2	• support classes within mainstream schools: classroom teacher (all subjects, including science)
Barry	58	Male	Masters of Special Education	12	• support classes within mainstream schools: classroom teacher (all subjects, including science)
Penny	37	Female	Bachelor of Science Graduate Diploma of Education	6	• School for Specific Purposes: science teacher
Sara	36	Female	Masters of Education	13	• School for Specific Purposes: classroom teacher (all subjects, including science)
Chloe	59	Female	Masters in Arts, Graduate Diploma of Special Education	28	• School for Specific Purposes: classroom teacher (all subjects, including science)
Betty	57	Female	Bachelor of ScienceGraduate Diploma of Education	22	• mainstream science classes: science teacher • support classes within mainstream schools: science teacher
Beck	35	Female	Bachelor of Science, Bachelor of Education	10	• mainstream science classes: science teacher • support classes within mainstream schools: science teacher
Rachel	56	Female	PhD Science	26	• mainstream science classes: science teacher
Sylvia	29	Female	Masters of Education (Biology/Chemistry majors)	8	• mainstream science classes: science teacher
Ben	29	Male	Masters of Teaching Bachelor of Medical science	7	• mainstream science classes: science teacher
Jessica	58	Female	Post graduate diploma in Education Bachelor of Science	25	• mainstream science classes: science teacher
Jack	62	Male	Diploma of Education Bachelor of Science	35	• mainstream science classes: science teacher
Kara	41	Female	Masters of Teaching Bachelor of Science (Biology)	12	• mainstream science classes: science teacher
Harriet	39	Female	Masters of Special Education	17	• mainstream science classes: Learning and Support teacher • support classes within mainstream schools: science teacher
Blake	36	Male	Masters of Special Education Bachelor Science and PDHPE education	12	• mainstream science classes: Learning and Support teacher • mainstream science classes: science teacher

### Interviews

The interview/focus group protocol (see Supplementary Information) was based on a literature review plus input from a multidisciplinary research team (bioethicist, researchers in health, special education and disability studies, genetics clinicians and educators). Eleven semi-structured interviews with individual teachers, and one focus group with four teachers, were audio and video-recorded via Zoom. Interviews and focus group were all about one hour long (range 45–60 min). Sessions lasted about one hour (range 45–60 min). Recordings were transcribed verbatim by a third-party provider.

### Data analysis

Interviews and focus groups were analysed using inductive content analysis [49]. Inductive rather than deductive content analysis was used based on the current lack of knowledge about this topic. During initial open coding, codes represented blocks of raw data. KMJ and IS independently open-coded each interview and resolved differences through discussion; SW coded two interviews. All codes were reviewed by KMJ and IS with disagreements resolved through discussion. Codes were clustered into categories by KMJ and discussed with JD, an experienced qualitative researcher[50]. Categories were then clustered into themes and sub-themes [44, 45]. Trustworthiness and validity were ensured through researcher triangulation [51], with two authors independently coding interviews and discussing results, and two senior authors overseeing data analysis. Peer-debriefing by experts in genetics and intellectual disability ensured research validity, with all authors engaged throughout the study.

## Results

**Fig. 1:**
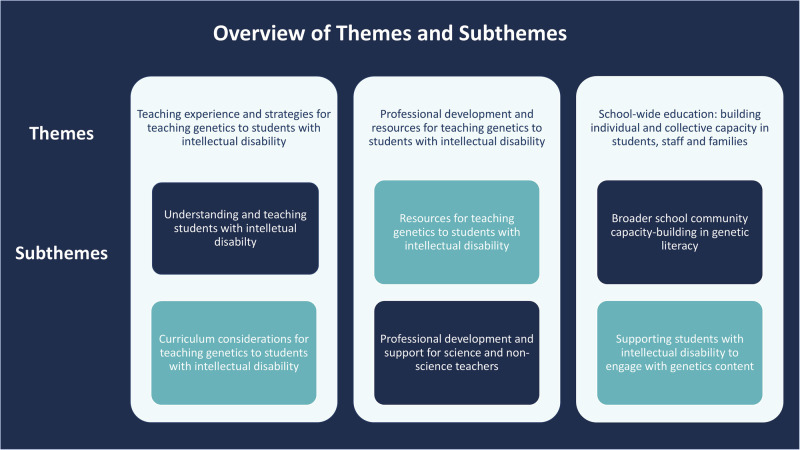
Overview of themes and subthemes. There are three themes, each of which has two subthemes, that show how research questions were answered by participants.

The results are organised into three main themes from the eleven interviews and single focus group (Fig. 1): (i) Teaching experience and strategies, (ii) Professional development and resources and (iii) School-wide education: building individual and collective capacity in students, staff and families. Each theme is broken into smaller subthemes to present key findings clearly.

### Theme 1: Teaching experience and strategies

This theme concerns participants’ experience teaching students with intellectual disability and/or genetics-related concepts, and strategies used to support curriculum access.

#### Understanding and teaching students with intellectual disability

This sub-theme explores the requirement for teachers to understand intellectual disability and use accessible, effective teaching approaches.

Foundational knowledge of intellectual disability is critical. Eleven participants had no tertiary-level special education training and five used outdated, problematic definitions, for example believing mild intellectual disability did not require diagnosis (Rachel, mainstream school [MS]), defining it as “…kids who struggle with processing information…” (Barry, specialist class in a mainstream school [SCMS]) or thinking “the reason they have an intellectual disability is because (…) things went wrong” (Harriet, MS). Teachers also lacked a strong knowledge of, or training in, evidence-based practices for teaching students with intellectual disability. Although most teachers reported using at least one evidence-based practice, such as visual supports, they had limited understanding of implementation and evaluation: “I know what to do in my bones, but I can’t tell you what it is” (Jack, MS)

Participants’ strategies for making genetic concepts, such as “identify that genetic information is transferred as genes in the DNA of chromosomes” [33] accessible for students with intellectual disability included simplified language and ‘message abundance’, presenting key ideas multiple times and ways according to student needs.

“Um, repetition? I guess it’s very multisensory… So, they’re visually seeing, hearing is auditory, they’re writing it, so whether it’s through fun games, whether it’s through song…” (Blake, MS)

Science-trained teachers showed a preference for “learning by doing”, stating students engage with experiential learning: “Giving them real life examples and applications, otherwise it doesn’t mean anything to the kids” (Rachel, MS). Non-science trained teachers prioritised functional skills: “I’m not expecting them to know the content. It’s the skills in accessing the content that is the focus” (Sara, specialist school (SS)), “….at least it means when they’re cooking with their parents, they can sort of participate a little bit” (Penny (SS)).

#### Curriculum considerations for teaching genetics to students with intellectual disability

Participants described differentiation strategies including identifying critical content “Kind of what they need to know, not just what would be nice to know” (Harriet, MS) and targeting student interests, “…look at where their interest lies and what stimulates their questions, their curiosity, and trying to get some basic skills” (Betty, MS). Other strategies included group work, hands-on learning, individual planning, building positive relationships and resource differentiation, “… the way we try to bridge it is by using whatever resources seem appropriate, but also bringing it back to the concrete…” (Jack, MS).

Participants acknowledged limited self-determination for students with disability “… in terms of the choices they can actually make, it’s quite limited…” (Ben, MS). However, they noted that ‘Life Skills’ courses supported student in-class decision-making, as additional time could address student interest: “…the flexibility of Life Skills outcomes (…) with the science syllabus, ….you’ve got to tick off the little things of what they’ve got to learn, rather than focus on something and they actually learn that particular thing really well and enjoy learning it” (Harriet, MS). However, non-science trained teachers could not identify which science Life Skills outcomes contained genetics content: “We kind of probably don’t realise that we’re actually teaching it (…) we don’t really make that connection in our head” (Sara, SS).

### Theme 2: Professional development and resources

This theme focuses on providing teachers professional development in teaching genetics that equips them with both essential knowledge and ready-to-use, pedagogically sound resources.

#### Resources for teaching genetics to students with intellectual disability

Teachers reported needing accessible genetics resources: “Teachers can’t teach [genetics] because of lack of resources, and the right resources for kids with intellectual disability” (Rachel, MS). Participants noted that student ability diversity meant that no single resource could meet all needs, emphasising the need for editable templates and customisable worksheets. They felt that textbooks sometimes use outdated and offensive language about disability and are inaccessible to students with intellectual disability: “.… have simplified science language in them [textbooks] so they’re accessible to all kids” (Blake, MS).

Teachers reported significant time demands for creating differentiated, strength-based resources: “The amount of time that we spend on writing the programs is not proportionate to the time that we have actually teaching them” (Sara, SS). Teachers reported using multiple sources, “…I was doing all of this myself, and finding resources online (…) YouTube (….) Khan Academy (…). I found TED Ed was the best resource (…), and then from that just trying to find lots of stuff. I even used stuff from university (…) Just hours and hours!” (Beck, MS) including online resources, notably subscription-based websites with downloadable materials.

Teachers requested visuals, songs and games, as well as developmentally appropriate videos, with presenters speaking slowly and using simplified English. “With the videos, it’s actually quite challenging to find ones that are pitched a suitable level” (Jack, MS). They also requested tangible DNA and chromosome models to present genetics-related vocabulary and content in multisensory ways:

“.…because if you show them this little model of a human, they may not necessarily relate …. having a life-sized one that you could open and say, “Look at all these things in here,” Yeah, that would be awesome.” (Sara, SS).

#### Professional development and support for science and non-science teachers

Teachers highlighted the critical gap in professional development for intellectual disability, science and genetic literacy - resources they consider essential for delivering potentially sensitive content safely. Confidence in teaching genetics reflected the variation in teacher preparedness. Five participants without science training reported having to teach high school science content (including genetics) to students with intellectual disability without having received specialised training or support. Teachers expressed concern about including content related to students’ disabilities, conditions in their families or genetic testing choices: “.…in reproductive technology you’re looking at amniocentesis…. That’s very sensitive” (Jessica, MS). Some confused genetic testing with genetic modification or therapy, with one participant referencing “about taking genes out, I suppose the dodgy genes and replacing them with the better genes” (Harriet, MS).

Teachers worried about potentially upsetting students with disability: “I’m more worried about inadvertently phrasing it in a way that makes the children anxious” (Penny, SS). However, one teacher viewed genetic literacy as vital for students who may later access genetic testing: “….at least they’ve had some preparation, and you just don’t know what difference it makes sometimes” (Sara, SS).

Some teachers also reported successfully speaking openly to students about disability, within a safe environment:

“…. where else would they get that information from? …. Why would you not just talk to them about all of these things? It’s really important. Especially getting into ethics…. And they ask questions….” (Candice, SCMS)

### Theme 3: School-wide education: building individual and collective capacity in students, staff and families

This theme explores the importance of building a school-wide understanding of disability and genetics and why genetic content is particularly important to students with intellectual disability.

#### Broader school community capacity-building

Participants raised the need to build understanding about intellectual disability and the value of genetic literacy for all members of the school community. This includes parents, “I think parent education is the key here” (Penny, SS), students with and without disability “…. kids in this school love to see the relevance of things…. So, if they know this is about explaining why someone is the way they are, they’ll be very interested to learn about it….” (Jack, MS), and teachers, “I’d love for more teachers to get involved, get more educated and access those skills, and maybe even professional learning” (Sylvia, MS).

#### Supporting students with intellectual disability to engage with genetics content

Thirteen teachers recognised genetic literacy in students with intellectual disability could be a “tool of empowerment and self-determination”, but this did not necessarily inform their current teaching. Three participants recognised the value of a genetic diagnosis to a person with intellectual disability, although knowledge about genetic testing varied: “Oh, my god, yes. I’ve never thought about that, to be honest with you” (Barry, SCMS).

“…First would be lack of teacher knowledge, so if – unless teachers are science-trained it can be quite hard, and, as you know, with a massive teacher shortage at the moment, you’re literally getting any man and his dog teaching any subject, so yeah, I guess it would be lack of teacher understanding of genetics themselves, and the other one is definitely, like, just the resources, having that ability to actually teach it effectively to people, (…) is it just a curriculum outcome, so, is it just a tick and flick, or is it something they’re actually going to spend time on and make sure the students thoroughly understand?” (Blake, MS)

Blake’s quote captures how effective teaching of genetics requires both teacher expertise and commitment - highlighting that educators must understand genetics and its importance before meaningfully teaching this critical knowledge that empowers students to make informed healthcare decisions.

## Discussion

### Teachers’ experiences

Although all participants taught genetics content to students with intellectual disability, their preparation and levels of confidence varied widely. Some had extensive experience while others did not realise that the science they teach is genetics, consistent with the international research literature [41, 52, 53].

In line with the preferences of interviewed adults with intellectual disability [3, 4] most participants (when directly asked) generally agreed genetic health content was important to teach. However, many had never considered the relevance to students with intellectual disability, and some expressed ethical concerns; thinking the content was too abstract for their students, and that building functional skills, such as cooking, was a better use of their time. Others worried that the students might be upset or triggered by these discussions. There are ongoing concerns about whether teachers are properly prepared to support students in these situations [54] given that most are neither trained psychologists nor genetics professionals.

Participants’ self-assessment that their genetic knowledge is outdated is also aligned with research [52, 55]. As previously reported by LaRue et al. [52] even teachers who knew genetics concepts had not been taught how to teach them, further diminishing teacher confidence. Teachers need to acquire pedagogical content knowledge (i.e., how to teach genetics) [37, 56] as part of their training and ongoing development. In addition, in line with Rafter and Gillies [57], teachers need to be aware of genetics’ potential significance for a person with intellectual disability. The link between the syllabus outcomes and students’ real-life experiences with genetic testing is rarely made explicit to teachers, although this has been recommended in the literature [53].

This study aligns with research findings about the lack of accessible resources on genetics for students with varied levels of support needs [11, 16], requiring teachers already under pressure to spend time creating accessible, differentiated resources. Consistent with international literature [20], most teachers were unaware that some of their pedagogical choices were in line with evidence-based practices for teaching students with disability. Although all participating teachers used visual supports, they only mentioned two of nine evidence-based practices (explicit instruction and manipulatives) that have been proven to improve academic outcomes for students with intellectual disability [19].

Our findings show a need for professional training in intellectual disability, genetics content and pedagogy, and understanding how genetics fits within the overall science syllabus.

### Enablers of school-based genetics education for students with intellectual disability

Consistent with findings from Cebesoy and Oztekin [15], we observed a relationship between teacher knowledge, confidence, and advocacy for genetic literacy teaching. Primary enablers include genetics content in syllabuses and teachers’ perception of its relevance for students with intellectual disability, which only two teachers with lived experience of genetic testing were able to articulate without prompting. Though thirteen teachers recognised genetic science literacy as a “tool of empowerment and self-determination”, none reported using self-determination interventions in their classrooms, such as goal setting and decision making, despite their effectiveness in improving academic outcomes [58] and building students’ skills in self-advocacy and problem solving [59]. None of their students were involved in lesson planning or individual learning planning, although three teachers reported building a sense of self-efficacy in their students, even if not formalised.

Participants suggested other potential enablers that aligned with national and international literature, including: support focused on curriculum outcomes together with high-quality, free, accessible and customisable resources for diverse audiences [11, 37]; a school-wide approach aligned with the DSE [29]; and teacher professional development in evidence-based practices supported by collaborative peer learning communities and peer coaching, as advocated by So et al. [60].

### Barriers

This study identified a wide range of barriers to school-based genetics education for students with intellectual disability. They include lack of accessible, flexible resources; siloed knowledge; and lack of clear supports and guidance. These combine to exacerbate the difficulty of teaching genetics within time constraints due to its complexity [11] and rapid pace of change [55]. Two other barriers relate to the nature of genetic content: sensitivity, and challenges for parents. In this study, teachers demonstrated a deep commitment to and care for their students (consistent with Rafter and Gillies [57]). However, this genuine care can become a barrier if teachers avoid educational content they perceive as sensitive, such as possible genetic causes of their students’ disabilities. Discomfort with genetic concepts and topics is widespread [61] and is consistent with findings from a recent study of the provision of sex education to students with intellectual disability in NSW schools in which a teacher reported avoiding topics she perceived as “too disturbing” for them [62]. The issue of sensitivity could be helped by greater student voice and agency in educational planning; in practice there is minimal inclusion, despite it being mandated by federal legislation [29].

Parental stress was mentioned as another barrier, acknowledging challenges caring for children with intellectual disability. Schools could utilise existing relationships to share appropriate resources and information. Trollor et al. [63] and Vissers et al. [64] found that families and individuals with intellectual disability who are genetically literate are more able to engage with and implement health interventions. Schools could capitalise on existing relationships and communication channels with parents to share appropriate resources and information such as directing them to websites such as the Centre for Genetics Education (https://www.genetics.edu.au).

### Teachers’ preferences for educational resources, supports and training

Participating teachers requested both training and resources about genetic content, and best practice pedagogy for students with intellectual disability, consistent with international research [16, 57]. Participants did not specify how professional training should be delivered, but several mentioned peer coaching, which has been shown to be effective [60]. Teachers would benefit from training in a range of evidence-based practices for students with intellectual disability which have also been shown to be effective for teaching science content. Some examples of these can be found in Table 2 [65, 66].

**Table 2:** Examples of evidence-based practices effectively used to teach science content to students with intellectual disability.

Researchers	Evidence-based practices and outcome
Knight et al. [65]	Combined graphic organisers, systematic instruction and task analytic instruction to teach scientific concepts
Courtade et al. [66]	Combined systematic instruction and the system-of-least-prompts to teach skills related to genomic literacy, such as heredity and cells
McKissick et al. [69]	Used video modelling, specifically slide shows which contain explicit instruction and videos, to teach science vocabulary
VanUitert et al. [70]	Used explicit instruction, via Content Acquisition Podcasts for Students (CAP-S), to support non-science trained teachers teaching science

Professional training and quality differentiated resources may provide more consistent experiences across school settings. Although all participants had similar requests, teachers in specialist schools may require more extensive support as they are usually not scientifically trained, teach multiple subjects, make extensive adjustments to resources, and may have limited access to science facilities or science-trained colleagues.

Whilst participating teachers did not indicate preferences for guidance materials on evidence-based practices, this may have been due to a lack of awareness [20]. They mentioned using evidence-based practices, particularly manipulatives, explicit and systematic instruction, and indicated interest in social narratives (brief, individualised descriptions of social situations) to support students with intellectual disability learn about genetic testing. Resources grounded in these types of evidence-based practices may give students with disability the individualised adjustments, supports and scaffolds they need to access genetic concepts more easily [19].

**Fig. 2:**
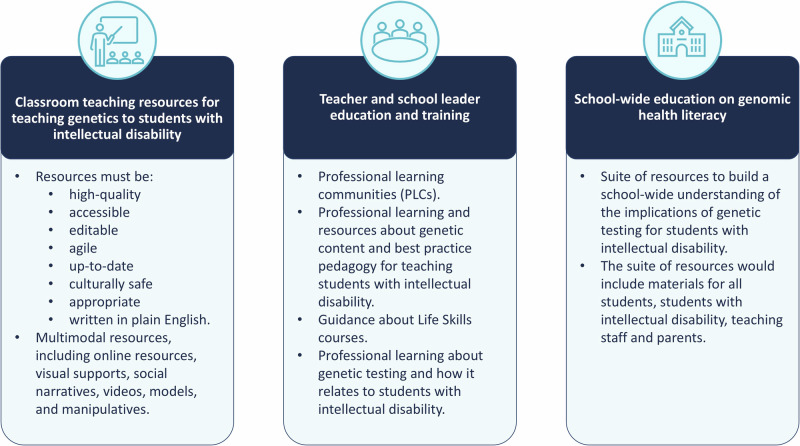
Summary of teacher requests. There are three main requests from teachers; resources, professional development and school-wide education on genomic health literacy. Detail is given below each request.

A summary of findings can be found in Fig. 2.

### Recommendations and practical implications of the study

A summary of recommendations, practical implications and recommendations for future research can be found in Table 3.

**Table 3:** A summary of recommendations, practical implications and recommendations.

Targeted stakeholder group	Recommendations for practice
Relevant state and federal education authorities	Language used in syllabus documents and teaching advice should be accessible and support the understanding of teachers teaching outside of their subject matter expertise.
	Teaching advice should refer to relevant evidence-based practices for students with disability.
	Create evidence-based, quality assured, accessible and editable student resources for teaching genetic content, with differentiated content to meet students’ diverse needs.
	Create professional learning about best practice pedagogy for teaching students with intellectual disability including awareness of ethical considerations and implications of teaching and/or discussing genetic and related health concepts.
Relevant state or federal health authorities	Create online resources for staff, students, and parents and families about genetic testing mapped to specific science syllabus outcomes.
	Co-design resources with students with intellectual disability and use co-production processes to assess relevance and social validity of these resources, such as an editable social narrative about genetic testing, with implementation guidance.
Universities	Pre-service teacher training should include a focus on intellectual disability, including how to use evidence-based practices.
	Science teacher training should include how to teach genetic concepts and related ethical considerations.

	Recommendations for future research
	This is the first known study into genetics teaching for students with intellectual disability in schools. Future research is recommended to capture broader experiences and teacher perspectives in different geographical and cultural contexts. Additional research should focus on evidence-based practices and their outcomes for students with intellectual disability. This includes research on the use of visual supports to meet the academic outcomes of students with intellectual disability and research on the use of a social narrative to build awareness of genetic testing for students with intellectual disability.

### Strengths and limitations

A key strength of this research is that it is the first known study to begin investigating how genetic content is taught to students with intellectual disability, by talking with teachers to determine what they do and what their concerns and thoughts are to support effective teaching. Participating teachers had varied teaching experience and qualifications and worked across the three main school settings in NSW that cater to students with disability. Several participants were very candid about not always meeting legislative requirements for students with disability, and reported feeling burned out and overwhelmed, suggesting low social desirability bias.

This research also has limitations. Participants were volunteers and so they may not be representative of all potential participants. Ensuring confidentiality and anonymity of participants partially mitigated the risk of volunteer bias.

## Conclusions

Genetic testing increasingly benefits healthcare for people with intellectual disability by guiding health surveillance and preventative measures to reduce premature mortality [67]. Genetic literacy, the capability to understand and apply genetic information to healthcare and decision-making [68], is therefore critical for people with intellectual disability for informed access to this information and improved long-term health and wellbeing outcomes.

Our findings suggest that if teachers understand the significance and relevance of genetics for students, they are better able to teach genetic content and encourage students with intellectual disability to build further genetic literacy. We identified significant barriers to supporting teachers developing genetic literacy in students with intellectual disability, including: a lack of available, customisable, accessible, culturally safe, and appropriate resources to address teachers’ concerns about sensitivity; professional development for teachers in genetic concepts and evidence-based practices to increase their confidence in supporting students with intellectual disability; and whole-of-school resources to support students, parents, caregivers and staff across the school. The practical challenges of teaching complex, changing content to students with intellectual disability are exacerbated by systemwide issues such as teacher shortages, that result in teachers with no tertiary qualifications in science teaching genetics. However, appropriate resources with teacher professional development may mitigate these barriers. This study will inform future approaches to resources and training, enabling teachers to empower students with intellectual disability to engage with genetic healthcare. Moreover, by supporting teachers to establish foundational genetic literacy in students with intellectual disability, we create opportunities for policymakers, health advocates and healthcare systems to co-design more effective and inclusive genetic healthcare initiatives.

## Supplementary information

Supplementary tables (1-6) and references

Interview protocol

Easy Read Summary of Article

## Data Availability

The datasets generated and/or analysed during the current study are not publicly available as transcripts could permit individual participants to be identified. However aggregate, non-identifying data is available from the corresponding author on request.
