# Supplementary material for: Developing genetic literacy in high school students with intellectual disability: Teachers’ experiences and perspectives
Source: Eur J Hum Genet. 2025 Jun 6;33(11):1530–8. doi: 10.1038/s41431-025-01865-2 (PMC12583577; doi:10.1038/s41431-025-01865-2)
Supplement: Supplementary file 1 — Supplementary tables (1-6) and references [file 41431_2025_1865_MOESM1_ESM.pdf]

Developing genetic literacy in high school students with intellectual disability: Teachers' experiences and perspectives

Karen-Maia Jackaman[1], Iva Strnadová[1,2,3], Sierra Angelina Willow[1], Julie Loblinzk Refalo[1,3], Jackie Leach Scully[2], Elizabeth Emma Palmer[4,5], Bronwyn Terrill[6,7]

Supplementary Tables

Supplementary Table 1

Some of the evidence-based practices proven to build learning and wellbeing outcomes for intellectual disability

Evidence-based practice	Research base
Embedded trial instruction	de Alvarenga et al., 2023 (1); Strnadová et al., 2023 (2)
Explicit instruction	Spooner et al., 2019 (3); Strnadová et al., 2023 (2)
Graphic organisers	Schnepel & Aunio, 2022 (4); Strnadová et al., 2023 (2)
Manipulatives	Bouck et al., 2018 (5); Long et al., 2022 (6); Spooner et al., 2019 (3); Strnadová et al., 2023 (2)
Modelling	Courtade et al., 2014 (7); Strnadová et al., 2023 (2)
Peer Support Arrangements	Brock & Huber, 2017 (8); Strnadová et al., 2023 (2)
Prompting	Courtade et al., 2014 (7); Strnadová et al., 2023 (2)
Reinforcement	Muharib et al., 2021
Response cards	Bondy & Tincani, 2018 (9); Clarke et al., 2016 (10); Strnadová et al., 2023 (2)
Response prompting strategies – including	Jimenez & Alamer 2018 (11); Schnepel & Aunio 2022 (4); Shepley et al., 2019 (12); Strnadová et al., 2023 (2)

constant and progressive time delay and system of least prompts	
Self-management	Briesch et al., 2016 (13); Smith et al.; 2022 (14)
Systematic instruction	Kuntz & Carter, 2019 (15); Strnadová et al., 2023 (2)
Task Analytic instruction	Spooner et al., 2019 (3); Strnadová et al., 2023 (2)
Video-based instruction and interventions	Allen et al., 2015 (16); Strnadová et al., 2023 (2); Wright et al., 2020 (17)
Visual supports / Visual activity schedules	Goo et al., 2019 (18); Malone et al., 2023 (19); Spriggs et al., 2017 (20); Strnadová et al., 2023 (2)

Supplementary Table 2: Science Years 7–10 Syllabus (2018), which includes Life Skills outcomes

**Relevant Life Skills outcomes:**	**Related Stage 4 outcomes:**	**Related Stage 5 outcomes:**
A student:<br><br>**SCLS-17LW:** recognises features of living and non-living things<br><br>**SCLS-18LW:** identifies structures of living things and their functions<br><br>**SCLS-19LW:** explores ways in which science and technology have improved human health	A student:<br><br>**SC4-14LW:** relates the structure and function of living things to their classification, survival and reproduction<br><br>**SC5-14LW:** analyses interactions between components and processes within biological systems	A student:<br><br>**SC5-14LW**: analyses interactions between components and processes within biological systems<br><br>**SC5-15LW**: explains how biological understanding has advanced through scientific discoveries, technological developments and the needs of society
**Life Skills genetic literacy specific content**	**Stage 4 genetic literacy specific content**	**Stage 5 genetic literacy specific content**
Students:<br><br>• investigate how scientific developments have changed or influenced the way people look after their bodies, eg the use of sunscreen to prevent sunburn, gym equipment to	Students:<br><br>• identify that living things are made of cells<br>• identify structures within cells, including the nucleus, cytoplasm, cell membrane, cell wall and chloroplast, and describe their functions	Students:<br><br>• identify that during reproduction the transmission of heritable characteristics from one generation to the next involves DNA and genes (ACSSU184)<br>• identify that genetic information is

exercise different parts of the body, refrigeration to store food, immunisation to prevent disease, or safety helmets and seatbelts<br>• communicate how advances in science and technology have improved our understanding of the causes and control of some infectious diseases	• outline the role of respiration in providing energy for the activities of cells<br>• identify that new cells are produced by cell division<br>• distinguish between unicellular and multicellular organisms<br>• identify that different types of cells make up the tissues, organs and organ systems of multicellular organisms<br><br>Students:<br><br>• research an example of how changes in scientific knowledge have contributed to finding a solution to a human health issue<br>• recount how evidence from a scientific discovery has changed understanding and contributed to solving a real world problem, eg animal or plant disease, hygiene, food preservation, sewage treatment or biotechnology	transferred as genes in the DNA of chromosomes<br>• outline how the Watson-Crick model of DNA explains:<br>– the exact replication of DNA<br>– changes in genes (mutation)<br>• describe, using examples, how developments in technology have advanced biological understanding, eg vaccines, biotechnology, stem-cell research and in-vitro fertilisation<br>• discuss some advantages and disadvantages of the use and applications of biotechnology, including social and ethical considerations<br><br>**Stage 5 Additional content**<br><br>Additional content is not prerequisite knowledge for

	• describe, using examples, how developments in technology have contributed to finding solutions to a contemporary issue, eg organ transplantation, artificial joints/limbs, treatment for diabetes, asthma, kidney or heart disease<br>• give examples to show that groups of people in society may use or weight criteria differently in making decisions about the application of a solution to a contemporary issue, eg organ transplantation, control and prevention of diseases and dietary deficiencies	following stages but may be used to broaden and deepen students' skills, knowledge and understanding in Stage 5.<br>Students:<br>• describe examples of advances in science and/or emerging science and technologies, in areas that involve biological science such as dentistry, environmental science, biomedical engineering, physiology, pharmaceuticals or nanotechnology<br>• assess the role of the development of fast computers in the analysis of DNA sequences<br>• research how information technology is applied in bioinformatics

Supplementary Table 3: Biology Stage 6 Syllabus (2017)

**Related Stage 6 outcomes: Year 11**<br><br>Module 1: Cells as the Basis of Life	**Related Stage 6 outcomes: Year 12**<br><br>Module 5: Heredity
A student:<br><br>**BIO11-8:** describes single cells as the basis for all life by analysing and explaining cells' ultrastructure and biochemical processes<br><br>**BIO11-9:** explains the structure and function of multicellular organisms and describes how the coordinated activities of cells, tissues and organs contribute to macroscopic processes in organisms	**BIO12-12:** explains the structures of DNA and analyses the mechanisms of inheritance and how processes of reproduction ensure continuity of species<br><br>**BIO12-13:** explains natural genetic change and the use of genetic technologies to induce genetic change

Stage 6 genetic literacy specific content

Cell Replication

Inquiry question: How important is it for genetic material to be replicated exactly?

- Students model the processes involved in cell replication, including but not limited to:
  - mitosis and meiosis (ACSBL075)
  - DNA replication using the Watson and Crick DNA model, including nucleotide composition, pairing and bonding (ACSBL076, ACSBL077)
- Students assess the effect of the cell replication processes on the continuity of species (ACSBL084)

DNA and Polypeptide Synthesis

Inquiry question: Why is polypeptide synthesis important?

- Students construct appropriate representations to model and compare the forms in which DNA exists in eukaryotes and prokaryotes (ACSBL076)
    - model the process of polypeptide synthesis, including: (ACSBL079)
    - transcription and translation
    - assessing the importance of mRNA and tRNA in transcription and translation (ACSBL079)
    - analysing the function and importance of polypeptide synthesis (ACSBL080)
    - assessing how genes and environment affect phenotypic expression (ACSBL081)
- investigate the structure and function of proteins in living things

Genetic Variation

Inquiry question: How can the genetic similarities and differences within and between species be compared?

- Students conduct practical investigations to predict variations in the genotype of offspring by modelling meiosis, including the crossing over of homologous chromosomes, fertilisation and mutations (ACSBL084)
- Students model the formation of new combinations of genotypes produced during meiosis, including but not limited to:
    - interpreting examples of autosomal, sex-linkage, co-dominance, incomplete dominance and multiple alleles (ACSBL085)
    - constructing and interpreting information and data from pedigrees and Punnett squares

- Students collect, record and present data to represent frequencies of characteristics in a population, in order to identify trends, patterns, relationships and limitations in data, for example:
    - examining frequency data
    - analysing single nucleotide polymorphism (SNP)

Inheritance Patterns in a Population

Inquiry question: Can population genetic patterns be predicted with any accuracy?

- Students investigate the use of technologies to determine inheritance patterns in a population using, for example: (ACSBL064, ACSBL085)
    - DNA sequencing and profiling (ACSBL086)
- Students investigate the use of data analysis from a large-scale collaborative project to identify trends, patterns and relationships, for example: (ACSBL064, ACSBL073)
    - the use of population genetics data in conservation management
    - population genetics studies used to determine the inheritance of a disease or disorder
    - population genetics relating to human evolution

Supplementary Table 4: Science Life Skills Stage 6 Syllabus (2017)

Note: The *Science Life Skills Stage 6 Syllabus* includes five courses (21):

- Investigating Science Life Skills
- Physical World Science Life Skills
- Earth and Space Science Life Skills
- Living World Science Life Skills
- Chemical World Science Life Skills.

**Related Stage 6 outcomes for Living World Science Life Skills course**<br><br>Module 3: Heredity and Genetics
**Content***: Inherited and Learned Traits*<br><br>**Inquiry question:** What is the difference between inherited and learned traits?<br><br>• Students recognise things that make people unique, for example: physical attributes, personality, likes and dislikes<br><br>• Students recognise traits as distinguishing characteristics ‡<br><br>• Students identify that traits can be visible and invisible<br><br>• Students classify personal traits as visible or invisible<br><br>• Students recognise that the way they look is different to others<br><br>• Students observe traits among a group of people, for example: gender, eye colour, hair colour, detached earlobes, tongue rolling, freckles, being right-handed, curly hair<br><br>• Students investigate the most common and least common traits of classmates by taking an inventory and recording data in a table or graph<br><br>• Students investigate traits shared by family members and record data

- Students use a model, for example a tree diagram, to demonstrate how traits are passed down from family members
- Students recognise that twins may or may not have the same traits
- Students recognise the transfer of traits from one generation to the next as heredity
- Students identify the difference between inherited traits and learned traits, for example:
    - physical attributes, eg eye colour and hair colour are inherited traits
    - favourite foods or sports may be learned traits
- Students explore the ways in which our cultural and social environments can lead to learned traits, for example, a favourite sport can come from living with family members who have a passion for that particular sport
- Students identify that living things are made up of cells that contain DNA
- Students recognise that deoxyribonucleic acid (DNA) provides the recipe for the traits inherited by a living thing
- Students recognise that all living things have both inherited and learned traits
    - Students investigate the inherited and behavioural traits of an animal species, for example, physical attributes of the red kangaroo, licking forearms on a hot day increases evaporation and cooling

Content*: Genetic Selection*

Inquiry question: How does an understanding of genetics impact on people's lives?

- Students explore how an understanding of human genetics has led to scientific advancements, for example: increased understanding of the link between genetics and diseases, eg cancer, heart disease and diabetes
- Students identify some ethical issues associated with an increased understanding of human genetics, for example: choosing gender for personal choice, reducing the chance of diseases or disabilities, creating donors for siblings
- Students explore examples of genetic selection in animals, for example:
  - selecting the temperaments of parent dogs for breeding dogs for different purposes, eg herding and guard dogs
  - using genetic selection to increase milk production in cows or wool production in sheep

Module 4: Disease and Disorders

Content

Infectious and Non-infectious Diseases and Disorders

Inquiry question: What is the difference between infectious and non-infectious disease?

- Students recognise common examples of diseases and disorders, for example: Influenza, measles, heart disease, diabetes, cancer
- Students recognise that infectious disease can be spread between people through direct contact or the air
- Students recognise that non-infectious diseases and disorders are caused by genetic, lifestyle or environmental factors
- Students classify a range of diseases and disorders as infectious or non-infectious

- Students explore why it is important to know whether a disease or disorder is infectious or non-infectious
- Students recognise that plants and animals can both be affected by disease

Content: *Technologies and Disorders*

Inquiry question: How can technologies be used to assist people with disorders?

- Students recognise a range of physical disorders, for example: sensory disorders, eg hearing and visual loss, cerebral palsy, muscular dystrophy, kidney or heart disorders
- Students engage with models of body parts to explore how they can be affected by a disorder, for example: ears, eyes, kidneys
- Students identify how technology can be used to assist with the effects of some disorders, for example: hearing aids and cochlear implants for hearing loss, spectacles and laser surgery for vision loss, dialysis for loss of kidney function

Supplementary Table 5

Inclusion and exclusion criteria for research participation

Inclusion criteria for teachers taking part in this study included:	Exclusion criteria for those who are not eligible to participate in the study were:
• High school teachers who have taught science to students with intellectual disability within the last ten years<br>• Learning and support teachers who have supported science teachers to teach science to students with intellectual disability within the last ten years<br>• Teachers currently working, or who have worked in NSW schools, including support units within mainstream schools and teachers in Schools for Specific Purposes (SSPs) within the last twelve months.	• High school teachers who have not taught students with intellectual disability<br>• Primary school teachers

Supplementary Table 6

Recruitment of participants and maintaining participant anonymity and data security

How recruitment occurred	Participants were recruited through social media advertisements, NSW Department of Education state-wide staffroom advertisements, and snowballing. As this is a convenience sample, it was not representative. The author emailed a soft copy of a recruitment flyer to potential participants. A link to Participant Information Statement and Consent form was attached to the email. The email also included direct project contact details.<br><br>Potential participants indicated their interest by contacting the author directly via email using the contact details available on the recruitment invitation. When a potential participant contacted the author, the author confirmed that they met the inclusion criteria provided also on the recruitment invitation. The author then undertook a consent process and arranged a time for the interviews. Consent forms were emailed to the author on or before the scheduled interview.
Withdrawal of consent	In the information sheet, participants were advised that they may withdraw at any time and how to do so.<br><br>Participants were told that their decision not to participate or to withdraw from the study would not affect their relationship with UNSW Sydney or any of the organisations involved in this research, the researchers would not collect additional information and that they could request that any identifiable information about themselves be withdrawn from the research project.<br><br>During the interview, the author checked again with the participant that they understood what participation entailed and reminded them that they

	were under no obligation to participate and that they could withdraw from the study at any time without penalty.
Maintaining anonymity and data security	Participants were given pseudonyms to ensure their anonymity.<br><br>The information about participants is stored in a re-identifiable format where any identifiers such as their name, age and gender will be replaced with a unique code.<br><br>Information collected from participants in an electronic format stored on a UNSW password protected OneDrive only accessible to the approved research investigators. Audio or video recordings stored on a UNSW password protected OneDrive are only accessible to the approved research investigators and a professional transcription service after a confidentiality agreement has been signed.

Supplementary References

1. de Alvarenga KAF, de Alcântara WL, de Miranda DM. What has been done to improve learning for intellectual disability? An umbrella review of published meta-analyses and systematic reviews. J Appl Res Intellect Disabil. 2023;36:413-28.
2. Strnadová I, Danker J, Dowse L, Tso M. Supporting students with disability to improve academic, social and emotional, and self-determination and life-skills outcomes: umbrella review of evidence-based practices. Int J Incl Educ. 2024;28:3606-3622.
3. Spooner F, Root JR, Saunders AF, Browder DM. An Updated Evidence-Based Practice Review on Teaching Mathematics to Students With Moderate and Severe Developmental Disabilities. Remedial Spec Educ. 2019;40:150-65.
4. Schnepel S, Aunio P. A systematic review of mathematics interventions for primary school students with intellectual disabilities. Eur J Spec Needs Educ. 2022;37:663-78.
5. Bouck EC, Park J. A Systematic Review of the Literature on Mathematics Manipulatives to Support Students with Disabilities. Education & Treatment of Children. 2018;41:65-106.
6. Long HM, Bouck EC, Kelly H. An Evidence-Based Practice Synthesis of Virtual Manipulatives for Students With ASD and IDD. Focus Autism Other Dev Disabl. 2023;38:147-57.
7. Courtade GR, Test DW, Cook BG. Evidence-Based Practices for Learners With Severe Intellectual Disability. Res Pract Persons Severe Disabl. 2014;39:305-18.
8. Brock ME, Huber HB. Are Peer Support Arrangements an Evidence-Based Practice? A Systematic Review. J Spec Educ. 2017;51:150-63.
9. Bondy AH, Tincani M. Effects of Response Cards on Students with Autism Spectrum Disorder or Intellectual Disability. Educ Train Autism Dev Disabil. 2018;53:59-72.
10. Clarke LS, Haydon T, Bauer A, Epperly AC. Inclusion of Students With an Intellectual Disability in the General Education Classroom With the Use of Response Cards. Prev Sch Fail. 2016;60(1):35-42.
11. Jimenez BA, Alamer K. Using Graduated Guidance to Teach iPad Accessibility Skills to High School Students With Severe Intellectual Disabilities. J Spec Educ Technol. 2018;33:237-46.

12. Shepley C, Lane JD, Ault MJ. A Review and Critical Examination of the System of Least Prompts. Remedial Spec Educ. 2019;40:313-27.
13. Briesch AM, Briesch JM. Meta-Analysis of Behavioral Self-Management Interventions in Single-Case Research. School Psych Rev. 2016;45:3-18.
14. Smith TE, Thompson AM, Maynard BR. Self-management interventions for reducing challenging behaviors among school-age students: A systematic review. Campbell Syst Rev. 2022;18:e1223-n/a.
15. Kuntz EM, Carter EW. Review of Interventions Supporting Secondary Students with Intellectual Disability in General Education Classes. Res Pract Persons Severe Disabl. 2019;44:103-21.
16. Allen KD, Vatland C, Bowen SL, Burke RV. An Evaluation of Parent-Produced Video Self-Modeling to Improve Independence in an Adolescent With Intellectual Developmental Disorder and an Autism Spectrum Disorder: A Controlled Case Study. Behav Modif. 2015;39:542-56.
17. Wright JC, Knight VF, Barton EE. A review of video modeling to teach STEM to students with autism and intellectual disability. Res Autism Spectr Disord. 2020;70:101476.
18. Goo M, Maurer AL, Wehmeyer ML. Systematic Review of Using Portable Smart Devices to Teach Functional Skills to Students with Intellectual Disability. Educ Train Autism Dev Disabil. 2019;54:57-68.
19. Malone K, Hollingshead A, Fodor J. Increasing Interaction in Social Settings for Students with Intellectual Disabilities Using Visual Supports. Educ Train Autism Dev Disabil. 2023;58:198-208.
20. Spriggs AD, Mims PJ, van Dijk W, Knight VF. Examination of the Evidence Base for Using Visual Activity Schedules With Students With Intellectual Disability. J Spec Educ. 2017;51:14-26.
21. Curriculum N. Science syllabuses: NSW Curriculum.https://curriculum.nsw.edu.au/learning-areas/science. Accessed 30 September 2024.
