# Supplementary material for: Developing genetic literacy in high school students with intellectual disability: Teachers’ experiences and perspectives
Source: Eur J Hum Genet. 2025 Jun 6;33(11):1530–8. doi: 10.1038/s41431-025-01865-2 (PMC12583577; doi:10.1038/s41431-025-01865-2)
Supplement: Supplementary file 2 — Interview protocol [file 41431_2025_1865_MOESM2_ESM.pdf]

Developing genetic literacy in high school students with intellectual disability: Teachers' experiences and perspectives

Karen-Maia Jackaman[1], Iva Strnadová[1,2,3], Sierra Angelina Willow[1], Julie Loblinzk Refalo[1,3], Jackie Leach Scully[2], Elizabeth Emma Palmer[4,5], Bronwyn Terrill[6,7]

Supplementary Material: Semi-structured interview protocol for teachers

Demographic questions

1. How old are you?
2. To which gender identity do you most identify?
3. What is your highest qualification?
4. How many years of teaching experience do you have? In what type of schools – mainstream, public, catholic/state/independent?
5. What is your school location?
    a. Sydney and surrounding suburbs
    b. Greater Sydney Metropolitan
    c. Regional NSW
    d. Rural NSW
6. What experiences do you have with teaching students with intellectual disabilities?
7. What (if any) training have you received on genetics science? In initial teacher preparation? In professional development?
8. Have you had any professional development in relation to intellectual disabilities and/or teaching genetics/ genomics to students with intellectual disabilities?

Questions: Curriculum considerations

1. Which NESA syllabus(es) are you using to teach content related to genetics? For example: Life Skills curriculum (7-10), The Living World Science Life Skills Stage 6 (Years 11-12), Science curriculum: (Years 7-10), Biology (Years 11-12).

    a. In what ways do you modify these?
2. When teaching a student with intellectual disabilities a Life Skills course, how do you select specific Life Skills syllabus outcomes?
3. In which ways do you involve the student and their parent/ carers within collaborative planning for learning?

Questions: Teaching strategies and practices

1. What approaches (teaching methods and strategies) do you use when teaching content related to genetics? PROMPT: using videos, guest speakers, visuals, explicit teaching…?
2. How do you build the field to help your students with intellectual disabilities understand the related vocabulary and concepts related to genetics?
3. What positive / negatives stories do you have to share about delivering content related to genetics to students with intellectual disability?
4. In what way, if any, are students with intellectual disability involved in determining what kind of content related to genetics they receive?

Questions: Resources

1. What type of resources have you used in teaching / supporting the teaching of content related to genetics?
2. Where did you access these resources from? PROMPT: Centre for genetics education, Australian Genomics, The Garvan Institute website. Colleagues, Delivery Support Staff, State Office staff/webpages, other
3. If I had a million dollars and a magic wand, what resources/training/tangible resources would I make for you?

   If clarification needed: What kind of resources, training, and support would you find helpful in developing your work from:

    a. Your school, for example your Head Teacher or Learning and Support Staff
    b. the Department of Education (State Office or Delivery Support)
    c. other agencies (for example the Council of Intellectual Disability or NSW Health)

Questions: Wrap up

1. How confident do you feel in teaching / support teaching of content related to genetics to students with intellectual disabilities?
2. To what extent do you think teaching / support teaching of content related to genetics to students with intellectual disabilities is important? If so, why / why not?
3. What do you think are two key barriers to teaching / support teaching of content related to genetics to students with intellectual disabilities?
4. What are potential challenges (and sensitivities) in resources related to genetics and genomics for students with intellectual disability?
5. Are there any questions about the topic we forgot to ask? Is there anything you would like to add?
