# Supplementary material for: Developing genetic literacy in high school students with intellectual disability: Teachers’ experiences and perspectives
Source: Eur J Hum Genet. 2025 Jun 6;33(11):1530–8. doi: 10.1038/s41431-025-01865-2 (PMC12583577; doi:10.1038/s41431-025-01865-2)
Supplement: Supplementary file 3 — Easy Read Summary of Article [file 41431_2025_1865_MOESM3_ESM.pdf]

Easy Read Version

What teachers think about teaching genetics to students with intellectual disability

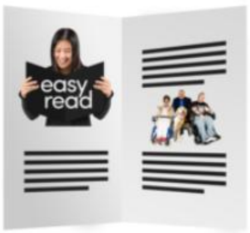

This booklet is a Summary of our **Research**

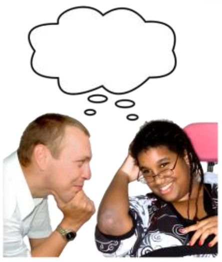

Research is something we do to find out

- what people think about something

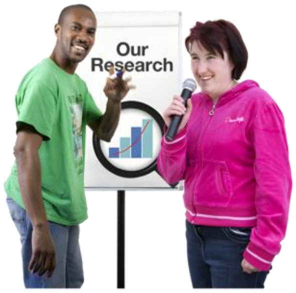

The full research is called

"Developing genetic health care literacy in high school students with intellectual disability: Teachers experiences and perspectives"

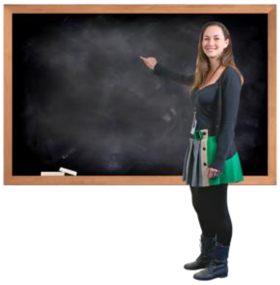

This research is about what teachers think

- about teaching **genetics**

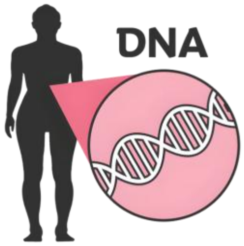

Genetics is the study of **genes**

- and how they work

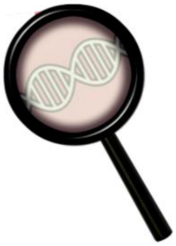

Genes are the things in your body

- that make you the way you are

What we know

People with intellectual disability

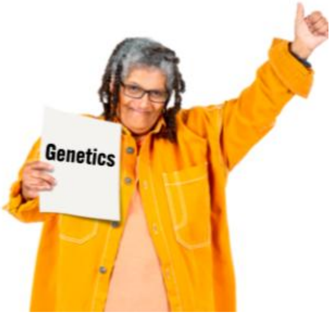

- have the right to accessible information
- want to know about genetics and
- wish they learnt about it at school

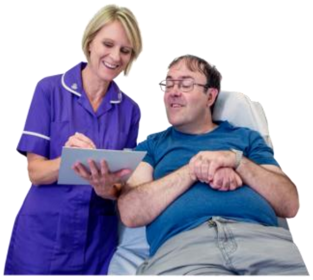

Knowing about genetics can make **genetic health care** better

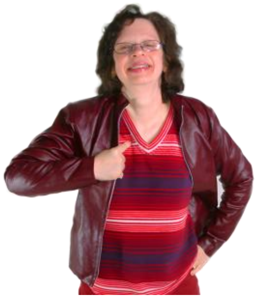

Genetic health care is for people who

- have a genetic condition
- might have a genetic condition
- want to know more about genetic conditions and tests

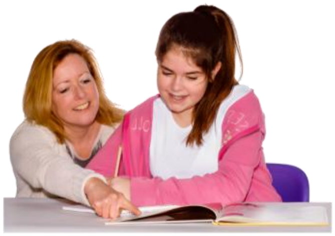

In school we can learn about genetics

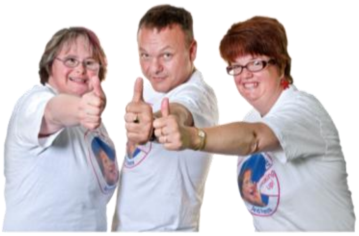

Learning about genetics can help people with intellectual disability

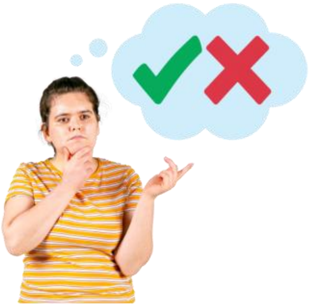

Knowing about genetics can help everyone

- to have choices and make decisions about their health

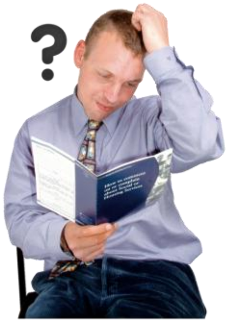

But teaching genetics is hard

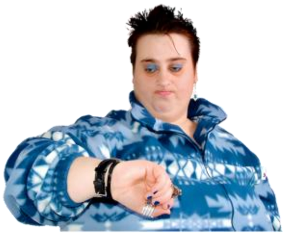

Students with intellectual disability need

- more time to understand information

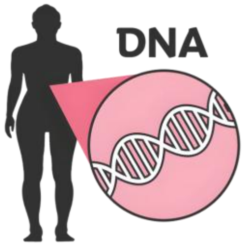

Sometimes teachers do not know about genetics

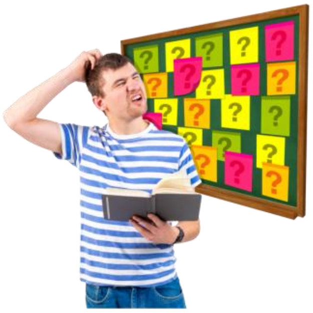

Sometimes teachers do not know

- how to teach students with intellectual disability

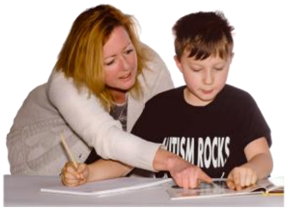

There are ways to teach students with intellectual disability

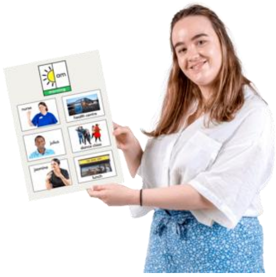

One example is **visual supports**

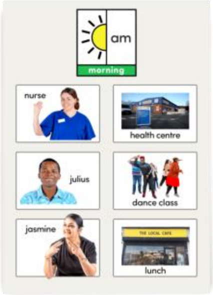

Visual supports are when pictures are used

- to help explain ideas

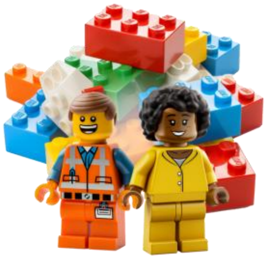

Another example is **manipulatives**

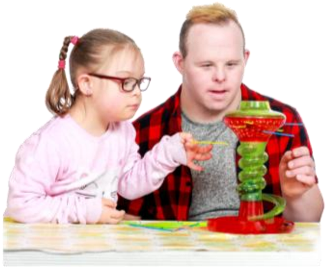

Manipulatives are things that students can

touch and move

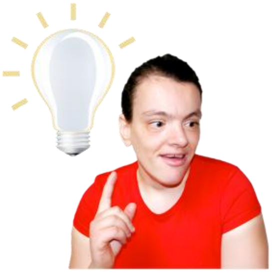

Manipulatives and visual supports

- help students understand
- ideas and
- problems

What we did

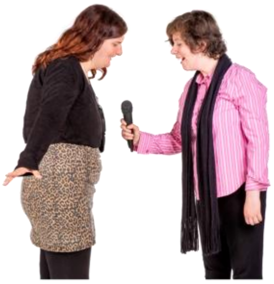

We talked to 15 teachers

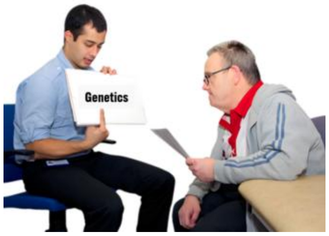

We asked them about teaching genetics

- to students with intellectual disability

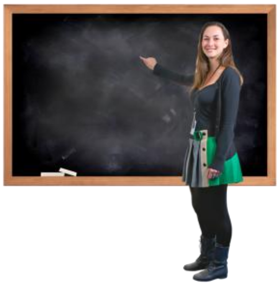

The questions were about

- their experience
- how they can do their job better
- why it is important to teach genetics

What we found out

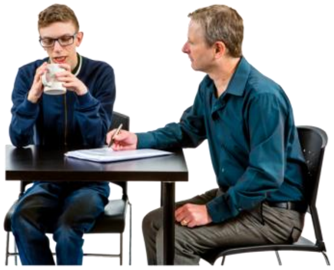

All teachers care about their students

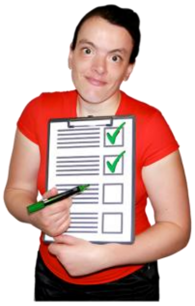

Some teachers told us good ways

- to teach genetics to students with intellectual disability

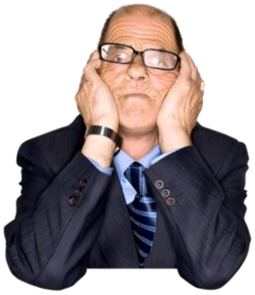

Teaching genetics to students with intellectual disability

- makes some teachers worried

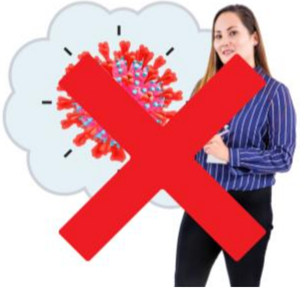

Some teachers are not Science teachers

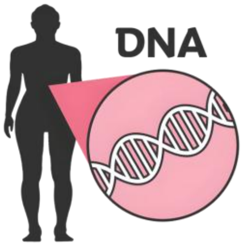

This makes it hard to teach genetics

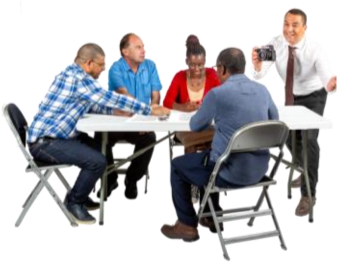

Teachers work hard to make resources

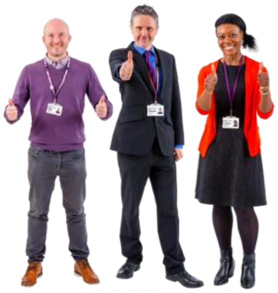

Teachers want good resources

How can teachers do their job better

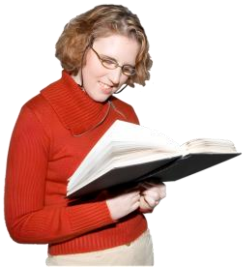

Teachers

- want to learn more about intellectual disability

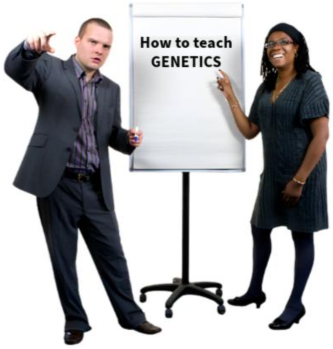

- need support to teach genetics

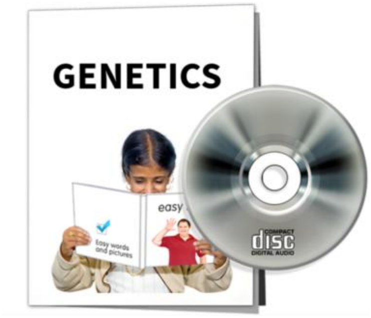

Support for teachers can be resources

- videos
- songs
- games
- worksheets

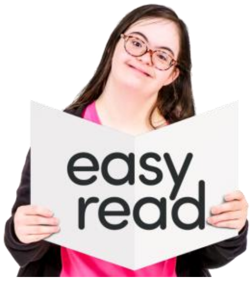

These resources need to be accessible

- for people with intellectual disability

Why teaching genetics is important

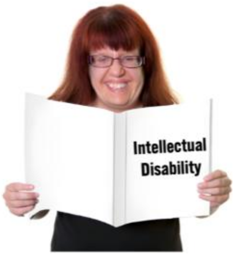

Teachers think everyone at school should

- learn about intellectual disability

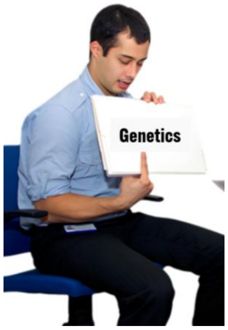

- learn about genetics

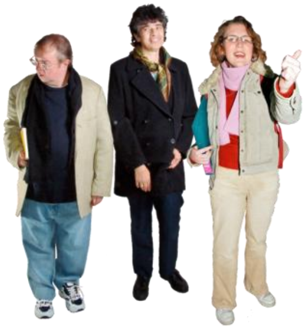

Everyone at school includes:

- teachers
- principals
- students and
- families

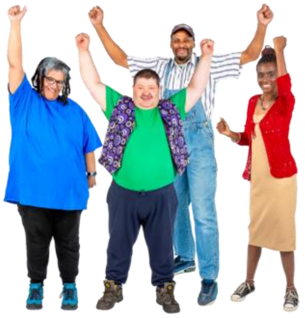

Learning about intellectual disability helps everyone

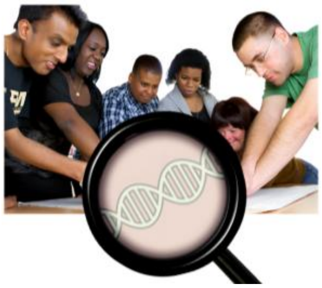

Learning about genetics helps everyone

How we can make things better

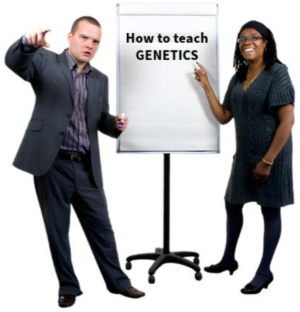

Teachers need training

- in genetics

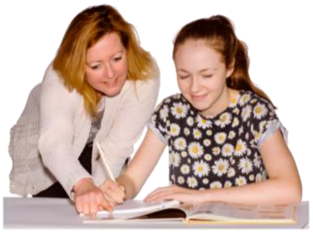

- how to teach students with intellectual disability

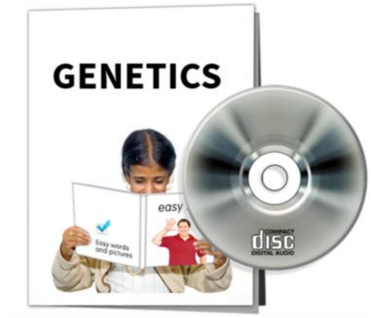

Teachers need good resources to teach students with intellectual disability

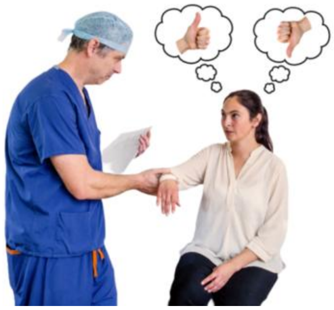

This can help students with intellectual disability

- make decision about their health

More information

made with photosymbols®

The GeneEQUAL team made this booklet

We use Photosymbols

The GeneEQUAL website has

- the full article and
- more information

Go to

www.genequal.com

or scan the QR code.
